# Evodiamine Attenuates P2X_7_-Mediated Inflammatory Injury of Human Umbilical Vein Endothelial Cells Exposed to High Free Fatty Acids

**DOI:** 10.1155/2018/5082817

**Published:** 2018-08-19

**Authors:** Yun Xue, Ting Guo, Lifang Zou, Yingxin Gong, Bing Wu, Zhihua Yi, Tianyu Jia, Shanhong Zhao, Liran Shi, Lin Li, Huilong Yuan, Hui Liu, Yun Gao, Guilin Li, Shuangmei Liu, Hong Xu, Chunping Zhang, Shangdong Liang, Guodong Li

**Affiliations:** ^1^Department of Physiology, Basic Medical College of Nanchang University, Nanchang, Jiangxi 330006, China; ^2^Jiangxi Provincial Key Laboratory of Autonomic Nervous Function and Disease, Nanchang, Jiangxi 330006, China; ^3^Clinic Medicine Department, Medical College of Nanchang University, Nanchang, Jiangxi 330006, China; ^4^Department of Cell Biology, Medical School of Nanchang University, Nanchang, Jiangxi 330006, China

## Abstract

Insulin resistance and type 2 diabetes mellitus (T2DM) are highly prevalent around the world. Elevated concentrations of free fatty acids (FFAs) are closely related to insulin resistance and T2DM. P2X_7_ receptor is an ion channel gated by ATP, which is implicated in various scenarios including immune response, pain, and inflammation. In this study, we have explored whether P2X_7_ receptor is involved in pathological changes in human umbilical vein endothelial cells (HUVECs) induced by high FFA treatment, and the potential beneficial effects of evodiamine. Evodiamine could effectively suppress the enhanced expression of P2X_7_ receptor caused by high FFAs at both mRNA and protein levels. In addition, high FFA-induced cytotoxicity, the upregulated release of ATP, and production of reactive oxygen species (ROS) could be ameliorated by evodiamine in HUVECs. Evodiamine could also reverse the decreased NO formation and the increased adhesive events of immune cells at high FFAs. Moreover, evodiamine inhibited P2X_7_-dependent TNF-*α* expression and ERK 1/2 phosphorylation due to high FFAs. All these results indicated that evodiamine could correct the upregulated expression of P2X_7_ receptor induced under high FFA condition in HUVECs, and consequently suppressed oxidative stress and inflammatory responses.

## 1. Introduction

Inflammation has been regarded as a risk factor for the development of insulin resistance and type 2 diabetes mellitus (T2DM) [1, 2]. Insulin resistance and T2DM are often accompanied by increased plasma levels of free fatty acids (FFAs), hyperinsulinemia, hyperglycemia, and atherosclerosis [3]. Vascular endothelial cells play an important role in vascular regulation, endocrine function, and maintaining cardiovascular homeostasis [4, 5]. Endothelial damage is a fundamental event for the development of atherosclerosis. In addition, the risk of vascular diseases is enhanced in the context of hyperinsulinemia, which also occurs in blood glucose poorly controlled diabetic patients [6]. Normal functions of endothelial cells are crucial to prevent insulin resistance or diabetes-induced large vessel atherosclerosis and the microvascular damage.

Adenosine triphosphate (ATP) can participate in the cellular signal transduction by binding to a class of P2X receptors, which are ligand-gated cation channels [7–10]. P2X_7_, a subtype of P2X receptors, plays an important role in inflammatory and immune responses. Uncontrolled Ca^2+^ influx may be induced due to the overstimulation of P2X_7_ receptor by extracellular ATP [11, 12]. ATP in the extracellular space can be increased after endothelial cells are damaged upon inflammation [13]. Moreover, high FFAs can enhance vascular insulin resistance by inhibiting insulin signaling [14, 15]. Many studies have found that P2X_7_ receptor mediates communications between neuron and microglia under inflammatory condition [16]. However, little is known about the effects of P2X_7_ receptor on human umbilical vein endothelial cells (HUVECs) under the pathological condition of high FFAs.

Evodiamine (EVO) is a natural alkaloid and found abundantly in fruits of *Evodia rutaecarpa*, a medicinal plant which has been used in Chinese medicine practice [17]. EVO has been shown to exhibit anti-inflammatory, antinociceptive, and anticancer activities [18–20]. However, the accurate mechanisms in improving inflammation by EVO remain little understood. Therefore, this study investigated whether P2X_7_ receptor participates in high FFA-induced endothelial dysfunction and EVO possesses potential protecting effects on vascular endothelial injury under high FFAs.

## 2. Materials and Methods

### 2.1. Materials

RPMI 1640 culture medium was purchased from Hyclone (USA); fetal bovine serum (FBS), from Biological Industries (Israel); EVO, from Nanjing Zelang Medical Technology, China; and 3-(4,5-dimethylthiazol-2-yl)-5-(3-carboxymethoxyphenyl)-2-(4-sulfophenyl)-2H-tetrazolium (MTS) assay kits, Revert Aid First Strand cDNA Synthesis Kit, and SYBR Green Master Mix, from Promega, USA. Nitric oxide (NO) assay kit (nitrate reductase method) and DCFH-DA were obtained from Nanjing Jiancheng Bioengineering Institute, China. BCECF-AM was purchased from Beyotime Biotechnology, China. TRIzol reagent was purchased from Tiangen, China. Polyvinyl difluoride (PVDF) membranes were purchased from Millipore (Bedford, MA, USA). HUVECs were purchased from CTCC Bioscience (Shanghai, China). THP-1 cells were obtained from Shanghai Institute of Cell Biology, Chinese Academy of Sciences.

### 2.2. HUVEC Culture

HUVECs were cultured in RPMI 1640 medium containing 10% FBS, 100 U/ml penicillin, and 100 mg/ml streptomycin sulfate in an incubator containing 5% CO_2_ at 37°C [5]. Cells were grouped into control (1% BSA), high FFAs (1 mM), control plus 0.25 *μ*M EVO, and high FFAs plus 0.25 *μ*M EVO. After the cells were seeded into six-well plates for 24 h, the media was replaced and added into 1 mM FFAs and 0.25 *μ*M EVO simultaneously. Then, HUVECs were incubated for 3 days. FFAs were a mixture of palmitate and oleate, 1 : 2 (*w*/*w*) [21]. During the treatment, the concentration of FBS in the medium was lowered to 2% to make cells in the quiescent state.

### 2.3. THP-1 Cell Culture

THP-1 cells, a human monocytic leukemia cell line, were maintained in culture in RPMI 1640 containing 10% of FBS and supplemented with 100 U/ml penicillin and 100 mg/ml streptomycin sulfate in a humidified circumstance at 37°C containing 5% CO_2_. The medium was replaced every two days.

### 2.4. Assessment of Cell Viability (MTS Assay)

Protective effects of EVO and cytotoxicity of different concentrations of FFAs (0.25, 0.5, 1, and 1.5 mM) on HUVECs were examined by MTS assays. HUVECs were seeded at 4500 cells/well in 96-well plates in a 200 *μ*l volume. After various treatments of HUVECs for 72 h, the medium was removed and 90 *μ*l serum-free medium and 10 *μ*l MTS reagent were added per well for 2.5 h incubation at 37°C. The absorbance of formed formazan was measured at 490 nm by a microplate reader (Sunrise, Tecan, Mannedorf, Switzerland).

### 2.5. Measurement of Total NO Synthesis

After the HUVECs were treated with different concentrations of FFAs or EVO for 72 h, the medium was collected for determining the concentrations of total NO using a nitrate reductase method according to the manufacturer's manual (Nanjing Jiancheng Bioengineering Institute, China). In this method, nitrate reductase turned NO-derived nitrate into nitrite, and thus, the nitrite concentrations in the culture media were assayed by measuring the absorbance of nitrite at 550 nm using a microplate reader. The calculation formula for NO concentrations is as follows: concentration of NO (*μ*M) = [((absorbance of treated wells − absorbance of blank wells)/(absorbance of standard wells − absorbance of blank wells)) × standard concentration (100 *μ*M)] × dilution folds of sample before measuring.

### 2.6. Determination of Intracellular ROS

Intracellular ROS production was examined by the DCFH-DA method [22]. HUVECs were cultured with control (1% BSA) or different concentrations of FFAs in the absence or presence of EVO on 24-well plates for 3 d. After washing the cells with phosphate-buffered saline (PBS) three times, the medium was replaced by serum-free medium containing 10 *μ*M DCFH-DA for 20 min incubation at 37°C to load the fluorescence dye. Then, cells were washed with PBS three times to remove the fluorescence probe not entering cells. Fluorescence was determined by a fluorescence plate reader (Tecan Infinite M200) at excitation and emission wavelengths of 485 and 535 nm, respectively.

### 2.7. Measurement of Extracellular ATP Release

ATP concentration in HUVEC supernatant was assessed using an ATPlite 1step kit (PerkinElmer Company) according to the manufacturer's protocol. Briefly, HUVEC supernatant was collected, respectively. Pure ATP stock was diluted to construct a standard curve of ATP concentrations: 7.81, 15.63, 31.25, 62.5, 125, 250, and 500 pM. Luciferase enzyme substrate solution (25 *μ*l) was added to each well of an enzyme-coated plate for incubation of 5 min. Subsequently, cell supernatant (25 *μ*l) was added to each well for 10 min. The results were gathered by measuring the intensity of luminescence generated by ATP-dependent luciferin-luciferase. The extracellular ATP levels were released relative to the control.

### 2.8. Cell Adhesion Analysis

THP-1 cells were loaded with 10 *μ*M BCECF-AM fluorescent probe for 30 min in the dark [23]. Labeled THP-1 cells were collected by centrifugation and washed three times with PBS. THP-1 cell suspension (0.2 ml) was added into the six-well plates containing attached HUVECs after different treatments for 3 d. After the two types of cells were cocultured at 37°C for 1 h, the plates were gently washed three times to remove nonadherent cells. The adhered fluorescent THP-1 cells were detected using fluorescence microscopy.

### 2.9. Real-Time RT-PCR

After various treatments, total RNA in cells was extracted by TRIzol Total RNA Reagent. 2 *μ*g RNA was reversely transcribed into cDNA using Revert Aid First Strand cDNA Synthesis Kit. Amplification reaction assays contained SYBR Green Master Mix and primers. GAPDH was used as the reference gene for normalization, and cDNA abundance was quantified by the ∆∆CT threshold cycle method. The sequences of primers were as follows: GAPDH, forward 5′TGACGTGGACATCCGCAAAG3′ and reverse 5′CTGGAAGGTGGACAGCGAGG3′, and P2X_7_, forward 5′-GAGTCCGAGGCAATCTAATG -3′ and reverse 5′-CTGTGATCCCAACAAAGGTC-3′.

### 2.10. Western Blotting

Cells were seeded in 6-well plates, followed by different treatments for 72 h. After that, protein was extracted in lysis buffer (RIPA : PMSF : PhosSTOP = 100 : 1 : 1) for 15 min on ice. The supernatant was collected. An equal amount of proteins of various samples were separated by 10% sodium deodecyl sulfate-polyacrylamide gel electrophoresis (SDS-PAGE) and transferred to PVDF membranes. The membranes were blocked using 5% nonfat dry milk at room temperature and then incubated with primary antibodies. After being washed three times with PBS, the membranes were incubated with horseradish peroxidase-conjugated secondary antibodies. Then, chemiluminescent signal was assessed by chemiluminescence development kit by an imaging system. The quantification of band intensity was performed by Image Pro-Plus software, and the expression levels of proteins were normalized to *β*-actin as the integrated optical density (IOD) ratio. The primary antibodies used are rabbit anti-P2X_7_ (1 : 200, Alomone Labs), anti-total ERK1/2 and anti-phospho-ERK1/2 MAPK (1 : 1000, Cell Signaling Technology), and anti-TNF-*α* (1 : 800, Abcam).

### 2.11. Statistical Analysis

All results were expressed as mean ± SEM, and SPSS 21.0 was used to perform the statistical analysis of data. One-way analysis of variance (ANOVA) followed by a post hoc Student's *t* test was used to determine the statistical significance. *p* < 0.05 is considered as significant difference.

## 3. Results

### 3.1. Protective Effect of EVO on HUVECs Cultured at High FFAs

**Figure 1:**
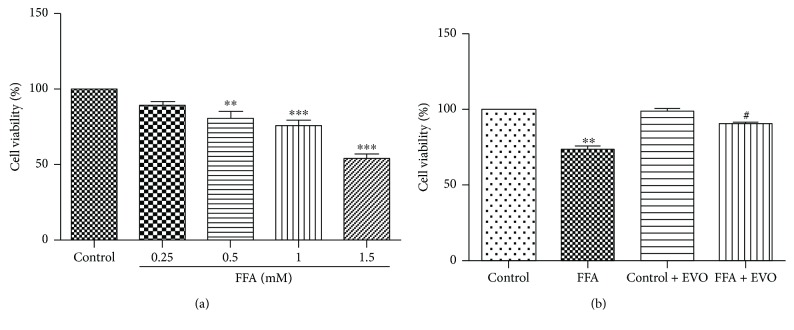
Effects of EVO on viability of HUVECs cultured with high FFAs. (a) Treatment of FFAs for 72 h reduced the cell viability in a dose-dependent manner. (b) Cells were cultured with control (1% BSA) or high FFAs (1 mM) in the presence or absence of 0.25 *μ*M EVO for 72 h. The cell viability was determined by MTS assay. The values are mean ± SEM of three independent experiments in triplicate. ^∗∗^*p* < 0.01 and ^∗∗∗^*p* < 0.001 versus control and ^#^*p* < 0.05 versus FFAs.

HUVECs were cultured in control (1% BSA) or different concentrations of FFAs for 72 h. The results show that FFAs affected cell viability in a dose-dependent manner (Figure 1(a)). A significant reduction of cell viability occurred upon treatment with 0.5, 1, and 1.5 mM FFAs. Exposure to high FFAs (1 mM) reduced the cell survival rate by 75% compared to the control group (Figure 1(b)). Meanwhile, the cytotoxic effect of high FFAs (1 mM) was abolished after coculture with 0.25 *μ*M EVO.

### 3.2. EVO Reversed the Effects of High FFAs on NO Formation

**Figure 2:**
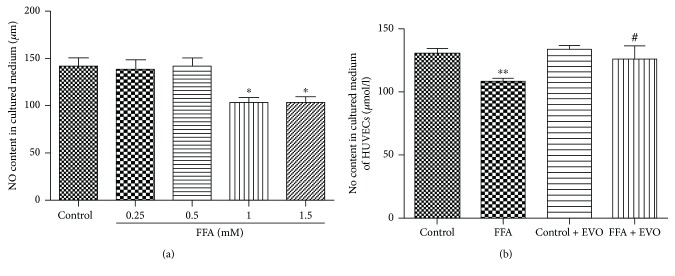
Effects of EVO on NO production in HUVECs. (a) High FFAs (1 and 1.5 mM) for 72 h could reduce the NO production. (b) HUVECs were cultured with control (1% BSA) or high FFAs (1 mM) in the absence or presence of 0.25 *μ*M EVO for 72 h. The values are mean ± SEM of three independent experiments in triplicate. ^∗^*p* < 0.05 and ^∗∗^*p* < 0.01 versus control group and ^#^*p* < 0.05 versus high FFA group.

Released NO levels in HUVECs were measured after 72 h treatment with 1% BSA, different concentrations of FFA, and 0.25 *μ*M EVO. The results showed that 1 and 1.5 mM FFAs reduced NO production (Figure 2(a)). High FFAs (1 mM) lowered NO formation by 17% (Figure 2(b); *p* < 0.01). However, coculture with EVO could bring NO content to the normal level. No significant change in NO production was seen when HUVECs were treated by EVO alone.

### 3.3. EVO Reduced the High FFA-Induced Increase of Intracellular ROS

**Figure 3:**
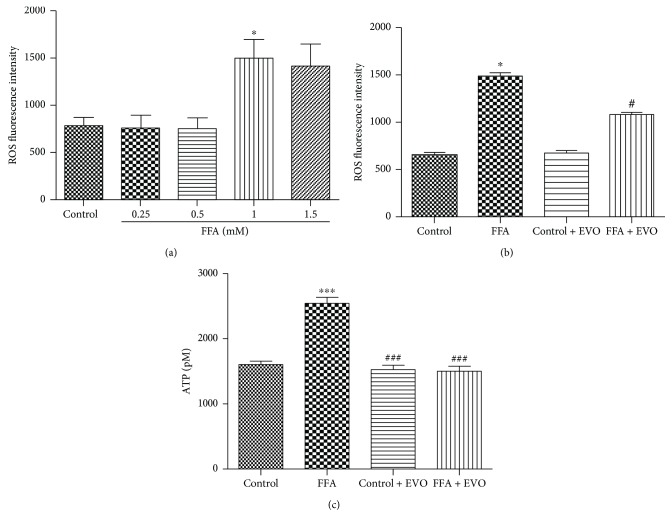
Effects of EVO on ROS production in HUVECs. (a) High FFAs (1 and 1.5 mM) increased ROS production. (b) HUVECs were cultured with control (1% BSA) or high FFAs (1 mM) in the absence or presence of 0.25 *μ*M EVO for 72 h. ROS production was examined after cells were loaded with 10 *μ*M DCFH-DA (a ROS fluorescence indicator). (c) Extracellular ATP in cell supernatant was significantly increased in HUVECs after the treatment of high FFAs. EVO could decrease ATP release in high FFA-treated cells. Data are mean ± SEM of three independent experiments in triplicate. ^∗^*p* < 0.05 and ^∗∗∗^*p* < 0.001 versus control group and ^#^*p* < 0.05 and ^###^*p* < 0.001 versus high FFA group.

ROS production in HUVECs was measured after 72 h treatment with 1% BSA, different concentrations of FFA, and 0.25 *μ*M EVO. The results revealed that 1 and 1.5 mM FFAs could increase ROS production (Figure 3(a)). Intracellular ROS generation was significantly increased in HUVECs after the treatment of high FFAs (1 mM) (Figure 3(b), *p* < 0.05). However, the enhanced NO production by high FFAs was mitigated in the presence of EVO.

### 3.4. Effects of High FFAs and EVO on Extracellular ATP Release

Extracellular ATP release was measured to further determine the effect of FFA and EVO on HUVECs. Extracellular ATP was significantly increased in HUVECs after the treatment of high FFAs (Figure 3(c), *p* < 0.001). However, EVO can decrease ATP release in high FFA-treated cells (Figure 3(c), *p* < 0.001).

### 3.5. Effects of High FFAs and EVO on P2X_7_ Receptor Expression

**Figure 4:**
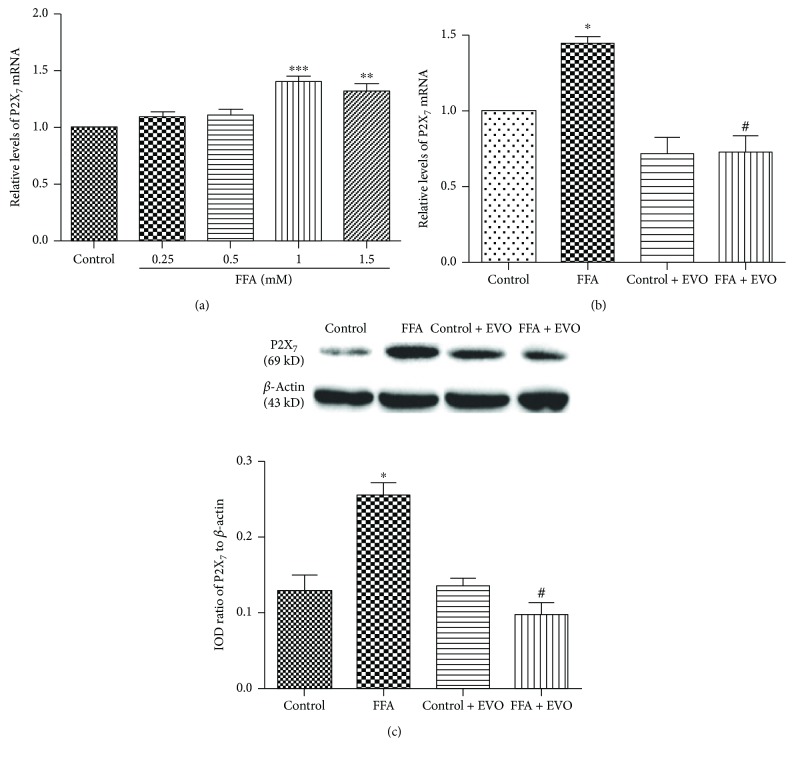
Effects of FFAs and EVO on P2X_7_ receptor expression in HUVECs. (a) 1 mM FFA had the most significant effect on elevating P2X7 mRNA level. (b) Cells were cultured with control (1% BSA) or high FFAs (1 mM) in the presence or absence of 0.25 *μ*M EVO for 72 h. The levels of P2X_7_ receptor mRNA were determined by real-time RT-PCR. (c) The P2X_7_ receptor protein was detected by Western blotting and quantified as the integrated optical density (IOD) ratio to *β*-actin. The values are mean ± SEM of three independent experiments in triplicate. ^∗^*p* < 0.05 versus control; ^#^*p* < 0.01 versus FFAs.

To explore the potential involvement of P2X_7_ receptor in FFA-induced adverse effects on HUVECs and how EVO protects HUVECs, the expression of P2X_7_ receptor at both mRNA and protein levels was detected. The result showed that 1 mM FFA had the most significant effect on P2X_7_ mRNA level (Figure 4(a)). The P2X_7_ receptor mRNA level in HUVECs treated with high FFAs was 3-fold higher than that in the control cells (Figure 4(b)). Western blotting proved the enhancement effect of high (1 mM) FFAs on P2X_7_ receptor (Figure 4(c)). Moreover, the high FFA-induced P2X_7_ expression was blocked when HUVECs were cotreated with 2.5 *μ*M EVO.

### 3.6. EVO Suppressed the High FFA-Induced Adhesion of THP-1 Cells to HUVECs

**Figure 5:**
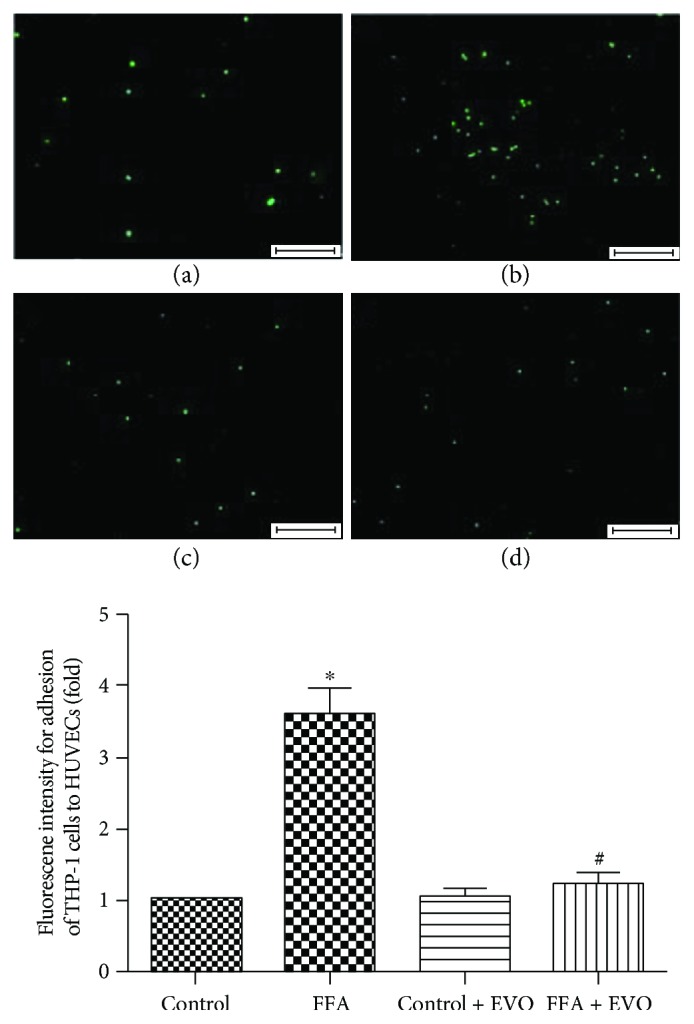
Effect of EVO on adhesion of THP-1 cells to HUVECs at high FFAs. HUVECs were cultured with control (1% BSA) or high FFAs (1 mM) in the absence or presence of 0.25 *μ*M EVO for 72 h. Adhesion was inspected after BCECF-AM-labeled THP-1 cells were cocultured with HUVECs for 1 h. The fluorescent cells in each well were counted, and the results in the histogram were expressed as the fold of the control. Data are mean ± SEM of three independent experiments in triplicate. ^∗^*p* < 0.05 versus control group and ^#^*p* < 0.05 versus high FFA group.

The adhesion of THP-1 cells to HUVECs cultured at high (1 mM) FFAs for 3 days was increased about 3.6-fold compared to control group (Figure 5), whereas 2.5 *μ*M EVO was able to suppress high FFA-induced adhesion of THP-1 cells to HUVECs. Thus, EVO effectively ameliorated the inflammatory reaction of HUVECs due to high FFAs.

### 3.7. Effects of High FFAs and EVO on ERK1/2 and TNF-*α* in HUVECs

**Figure 6:**
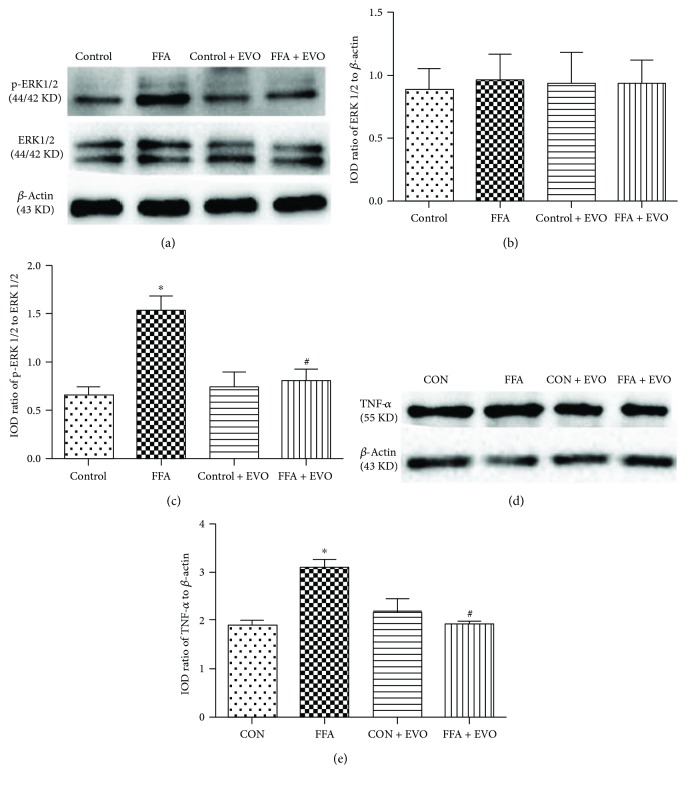
Effects of EVO on the levels of ERK 1/2, p-ERK 1/2, and TNF-*α* in HUVECs. HUVECs were treated with control (1% BSA) or high FFAs (1 mM) in the absence or presence of 0.25 *μ*M EVO for 3 d. (a) Gel images of Western blotting show the expression of ERK 1/2 and p-ERK 1/2. (b) Bar graph indicates the IOD ratio of ERK 1/2 to *β*-actin. (c) Bar graph indicates the IOD ratio of p-ERK 1/2 to ERK 1/2. (d) Western blotting images show the expression of TNF-*α*. (e) Bar graph indicates the IOD ratio of TNF-*α* to *β*-actin. The data are mean ± SEM of three independent experiments in triplicate. ^∗^*p* < 0.05 versus control group and ^#^*p* < 0.05 versus high FFA group.

**Figure 7:**
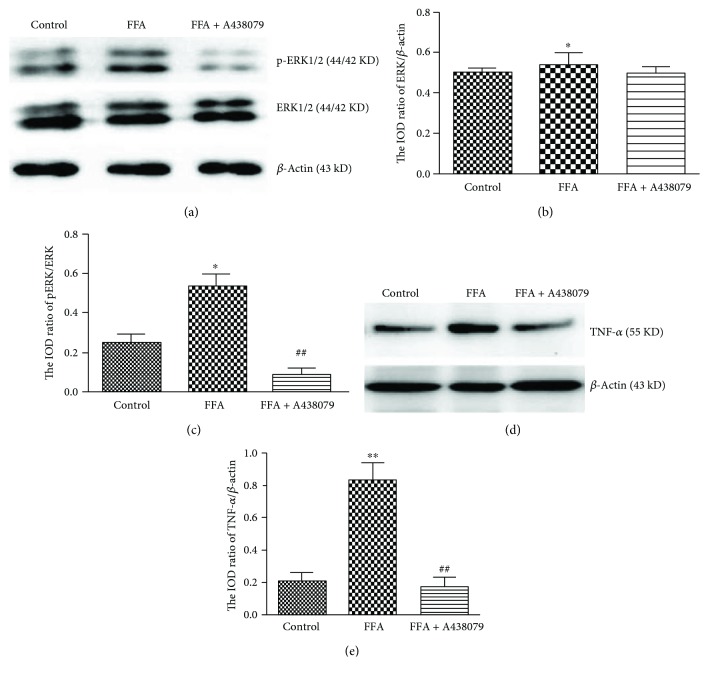
A438079 inhibited ERK 1/2, p-ERK 1/2, and TNF-*α* expression in HUVECs. HUVECs were treated with control (1% BSA) or high FFAs (1 mM) in the absence or presence of 10 *μ*M A438079 for 3 d. (a) Western blotting images show the expression of ERK 1/2 and p-ERK 1/2. (b) Bar graph indicates the IOD ratio of ERK 1/2 to *β*-actin. (c) Bar graph indicates the IOD ratio of p-ERK 1/2 to ERK 1/2. (d) Gel images show the expression of TNF-*α*. (e) Bar graph indicates the IOD ratio of TNF-*α* to *β*-actin. The data are mean ± SEM of three independent experiments in triplicate. ^∗^*p* < 0.05 and ^∗∗^*p* < 0.01 versus control group and ^##^*p* < 0.01 versus high FFA group.

The expression and activity of ERK1/2 and TNF-*α* in HUVECs were assessed by Western blotting. The ratio of pERK1/2 to ERK1/2 in the high FFA group was about 2.5-fold higher than that in the control cells (Figures 6(a) and 6(c)), and the ratio of ERK1/2 to *β*-actin was not different between the two groups (Figures 6(a) and 6(b)). In addition, cotreatment of EVO was able to reverse high FFA-induced activation of ERK1/2 in HUVECs (Figures 6(a) and 6(c)). Similarly, the expression of TNF-*α* was also increased in the high FFA group compared to the control group, which could be suppressed when cells were cocultured with EVO (Figures 6(d) and 6(e)). To define if the effects of high FFAs on p-ERK and TNF-*α* are P2X_7_-dependent, 10 *μ*M A438079 (a selective inhibitor of P2X_7_) was used. The results showed that p-ERK and TNF-*α* were reduced in the cells treated with FFA + A438079 compared to the treatment by FFAs only (Figure 7).

## 4. Discussion

Vascular endothelial dysfunction occurs in insulin resistance, diabetes, and other metabolic diseases [24]. Recent studies have found that concentrations of plasma FFAs were increased under the condition of impaired endothelial dysfunction and insulin resistance, suggesting that high FFAs had some adverse effects on endothelial cells [25, 26]. Published studies also revealed that reduced vascular NO bioactivity and endothelial dysfunction would occur when superoxide production was increased in experimental models of diabetes [27]. P2X_7_ receptor expression was upregulated in inflammatory and apoptotic responses in endothelial cells treated with high glucose in vitro [28]. However, the relationship between P2X_7_ receptor and high FFAs is not clear. In this study, chronic exposure of HUVECs to FFAs affected cell viability in a dose-dependent manner. Moreover, we found that P2X_7_ expression was increased under high FFA condition and EVO could protect HUVECs from high FFA-evoked viability reduction, suggesting a potential role of P2X_7_ and EVO in FFA action on endothelial cells.

NO plays an important role in mediating a variety of biological functions, including immunity, vasodilation, neurotransmission, and vascular permeability. However, NO-mediated vascular relaxation was damaged in insulin resistance and diabetes, suggesting endothelium dysfunction [29]. Furthermore, endothelial cell injury and dysfunction are associated with upregulation of oxidative stress. Increased ROS in vivo due to too much ROS production or reduced antioxidant capacity can lead to oxidative injury to cells. It is also well known that endothelial NO can suppress vascular inflammation by inhibiting the adhesion of immune cells to endothelium. In our study, NO formation was dramatically decreased whereas ROS production was increased in HUVECs under high FFA condition. In addition, ATP released from damaged cells may act as an inflammatory mediator to participate in inflammation [30, 31]. We observed that treatment of high FFAs led to the increased release of extracellular ATP from HUVECs. EVO could antagonize these effects of high FFAs on NO, ROS, and ATP production in HUVECs, indicating its strong capability of antioxidative stress, anti-inflammation, and improving endothelial function. Insulin resistance and diabetes are often accompanied with mild inflammation and associated with changes in gene expression [32, 33]. Abnormal expression of P2X_7_ receptor is involved in inflammation, and P2X_7_ receptor plays a critical role in the regulation of glial cells [34]. Upregulated expression of both P2X_7_ mRNA and protein was observed in the current study after HUVECs were exposed to high FFAs. Thus, our results suggested that P2X_7_ receptor might be implicated in the abnormal changes in production of NO, ROS over the course of inflammation, and vascular endothelial dysfunction induced by high FFAs. EVO can decrease production of ROS and ATP to protect cells.

We assessed the adhesion of THP-1 cells to HUVECs, because of the key role of increased mononuclear-endothelial cell interactions in insulin resistance- or diabetes-induced inflammatory reaction [35]. Our results showed that the adhesion of THP-1 cells to HUVECs was strengthened after HUVECs were pretreated with high FFAs. Such upregulated adhesion was markedly blocked by EVO, implying its strong ability to inhibit inflammatory reaction. Inflammation may involve increased expression and activity of certain intracellular signaling pathways. Increased P2X_7_ receptor may activate ERK 1/2, which leads to phosphorylation of ERK 1/2. TNF-*α* is an underlying proinflammatory cytokine implicated in diverse pathological processes [36]. Our results indicated that activation of ERK 1/2 was increased in high FFA-cultured HUVECs, suggesting that ERK1/2 might play an important role in the regulation of high FFA-induced expression of inflammatory cytokines such as TNF-*α* and IL-1*β*. In addition, EVO was able to suppress high FFA-induced phosphorylation of ERK 1/2 and the increased expression of TNF-*α* in HUVECs in P2X_7_-dependent manner. Therefore, the activation of ERK 1/2 signal pathway might participate in the molecular mechanism for P2X_7_-mediated TNF-*α* release and inflammation in high FFA-cultured HUVECs.

In conclusion, HUVECs cultured with high FFAs exhibited an increased expression of P2X_7_ receptor whereas EVO was able to counteract this effect. Furthermore, the exposure of HUVECs to high FFAs resulted in decreased cell viability and NO content, enhanced ROS production, and upregulated ERK 1/2 phosphorylation and TNF-*α* expression. In contrast, EVO could reverse these harmful outcomes of high FFAs probably mainly by acting on the blockade of P2X_7_ activity. Therefore, EVO protected endothelial cell function from high FFAs by anti-inflammatory and antioxidative effects.

## Data Availability

The data used to support the findings of this study are available from the corresponding author upon request.
